# Cutaquig^®^ Is Well Tolerated in Immunodeficient Patients Who Did Not Tolerate Other Subcutaneous Immunoglobulin Products

**DOI:** 10.3390/hematolrep14040048

**Published:** 2022-11-17

**Authors:** Sydney Brownlee, Crystal Allen, Mohammed F. Kana’an, D. William Cameron, Juthaporn Cowan

**Affiliations:** 1 Clinical Epidemiology Program, The Ottawa Hospital Research Institute, Ottawa, ON K1H 8L6, Canada; 2 Department of Nursing, The Ottawa Hospital, Ottawa, ON K1H 8L6, Canada; 3 Division of Infectious Diseases, Department of Medicine, University of Ottawa, Ottawa, ON K1H 8L6, Canada; 4 Centre of Infection, Immunity and Inflammation, Faculty of Medicine, University of Ottawa, Ottawa, ON K1H 8M5, Canada

**Keywords:** hypogammaglobulinemia, immunoglobulin treatment, subcutaneous immunoglobulin, tolerability, Cutaquig^®^

## Abstract

Objective: Subcutaneous immunoglobulin (SCIG) treatment is generally tolerable, but some patients may experience adverse events to one or more SCIG products. We investigated whether 16.5% Cutaquig^®^ treatment offered a tolerable and safe alternative treatment for immunodeficient patients. Methods: A one-year prospective cohort study was conducted at a single center in Ottawa, Canada. Adult immunodeficient patients who reported previous intolerability, adverse events, or other difficulty to other 20% SCIG product(s) were recruited to start on 16.5% Cutaquig^®^. Treatment tolerability, safety, and quality of life were observed and described. Results: Seven out of ten patients tolerated Cutaquig^®^. There were no serious or severe adverse events related to the treatment. Three moderate infections were reported (two urinary tract infections and one injection site infection). The mean serum IgG level at the end of the study was comparable to baseline levels recorded before the study: 9.6 ± 4.5 vs. 7.6 ± 4.3 g/L, *p* = 0.07. The overall health and health domain changes in the SF-36 and quality of life tests using the EQ visual analog scale improved by 21.5% (*p* = 0.38), 16.7% (*p* = 0.29), and 7.7% (*p* = 0.23), respectively. Conclusions: Cutaquig^®^ may be used as an alternative treatment option for patients who did not tolerate 20% SCIG products.

## 1. Introduction

Immunoglobulin (Ig) replacement therapy either via the intravenous or subcutaneous route is the mainstay treatment for patients with immunodeficiencies [1]. Subcutaneous immunoglobulin (SCIG) is well-tolerated and effective treatment preferred by many patients and their families due to the low incidence of systemic adverse events, increased ease of infusion, autonomy, and consistent steady-state pharmokinetics [1,2,3].

Local reactions at the infusion site are common with SCIG, but are typically mild, decrease over time, and do not impede the tolerability of treatment [4]. Nevertheless, some patients demonstrate poor tolerability to available SCIG products via persistent adverse events worsening with subsequent infusions. Common adverse effects of existing SCIG products include fatigue, headache, infusion site pain, and erythema [4]. In Canada, Hizentra^®^ (20% SCIG) was the first approved SCIG product. As of 2018, Cuvitru^®^ (20% SCIG) and Cutaquig^®^ (16.5% SCIG) were approved by Health Canada and made available by Canadian Blood Services. Compared with other commercially available 20% SCIG products, Cutaquig^®^ demonstrates a lower viscosity [5]. This is a valuable feature that may allow for an improved infusion experience for patients that did not tolerate 20% SCIG products [5].

This study aims to evaluate the tolerability of 16.5% Cutaquig^®^ in patients unable to tolerate 20% SCIG products. 

## 2. Methods

### 2.1. Study Design and Setting

This is a prospective cohort study before and after change in treatment formulation. The eligibility criteria included adult patients with PID or SID who were undergoing SCIG treatment. The participants must have developed adverse events or other difficulties in response to their SCIG treatment and were willing to change the treatment product. The patients were recruited at the Ottawa Hospital, Ontario, Canada from September 2018 to September 2020. A written informed consent was obtained from each patient prior to study enrolment. 

All patients received weekly subcutaneous Cutaquig^®^ infusions, starting at their last previous SCIG dosage and infusion frequency. The patients/caregivers received training either by a pump or push method at the study site to self-administer Cutaquig^®^ for their first infusion to ensure proper administration technique. All subsequent infusions were self-administered by the patients at home. 

### 2.2. Assessments

The patients participated in routine visits to the study site at 12-week intervals for a total of 48 weeks. The patients were required to maintain a journal documenting parameters related to infusion, including infusion date, infusion start and stop time, method of infusion (push vs. pump), volume, and location of infusions as well as any adverse events. 

Additionally, patient’s qualities of life (QoL) were assessed at each visit using the 36-item Short Form Health Survey (SF-36), the European Quality of Life Five Dimension Questionnaire (EQ-5D-5L), and the EQ Visual Analog Scale (EQ-VAS). The QoL data (SF-36 and EQ-5D-5L) were analyzed at the baseline and week 48, whereas the EQ-VAS data were analyzed at each 12-week interval. 

The adverse events (AEs) and tolerability, including infusion site reactions, were recorded throughout the study period. The AEs were classified as mild (discomfort noticed but no disruption of normal daily activity), moderate (discomfort sufficient to reduce or affect daily activity), severe (inability to work or perform normal daily activity), and serious (immediate threat to life or death). The patients were considered to tolerate Cutaquig^®^ if they were able to continually use the product without having to stop infusions or switch products.

Blood samples were collected from each patient at the baseline, just before Cutaquig^®^ was started, and at the week 48 visit, when possible, to test the blood concentration of immunoglobulins. 

The data were descriptively analyzed. The statistical analyses were performed for comparisons of QoL scores between the baseline and at the end of the study. A two tailed t-test was used for continuous variables. The missing data were excluded from the statistical analyses. The data used for the analysis can be found in the Supplementary Material File. 

## 3. Results

### 3.1. Study Population

A total of 10 patients were recruited in the study (Table 1). The median age of patients was 50 years (range: 31–73 years) and 70% (*n* = 7) were female and 30% (*n* = 3) were male. Six and four were diagnosed with PID and SID, respectively.

Just prior to the start of the study, most patients were being treated with Cuvitru^®^ (*n* = 8, 80%), whereas one patient was being treated with Hizentra^®^ (10%) and one with Gammagard^®^ (10%). All patients had been previously treated with either Cuvitru^®^ or Hizentra^®^ and 70% of patients had been treated with both. Other IVIG products used to previously treat patients include Gammunex^®^ and Panzyga^®^.

The most common AEs reported by patients from previous SCIG treatments include erythema (*n* = 4, 40%), headache (*n* = 3, 30%), and fatigue (*n* = 3, 30%).

### 3.2. Safety and Tolerability

Overall, seven (70%) completed the study without any administration changes, such as slowing, interrupting, or stopping the infusion. Three patients discontinued the use of Cutaquig^®^ due to AEs (restless leg associated with infusions, headache, fatigue, redness, and worsening itchiness at the injection site. Table 2). All patients had reported similar AEs during treatment with previous SCIG products. 

There were no severe or serious AEs related to IgG treatment reported in the study. Overall, eight treatment related AEs were reported in 6 out of 10 patients. All related local AEs were mild (*n* = 4, 50%) or moderate (*n* = 4, 50%). The most common reactions were infusion site erythema (*n* = 3, 37.5%) and fatigue (*n* = 3, 37.5%). Three moderate infections were reported (two treatment unrelated urinary tract infections and one treatment related injection site infection requiring treatment at the emergency department). There was no pneumonia, bacteremia, or any infections requiring hospitalization.

### 3.3. Infusion Parameter at Week 48

At week 48, the last study visit, most patients used pump-assisted SCIG infusion (Table 2). Only one patient infused SCIG using a push method. This patient required the lowest Ig dosage per week. The number of infusion sites ranged from 1 to 4, once or twice a week. The average infusion time was 70.8 min. 

### 3.4. Patient Satisfaction and Quality of Life 

**Figure 1:**
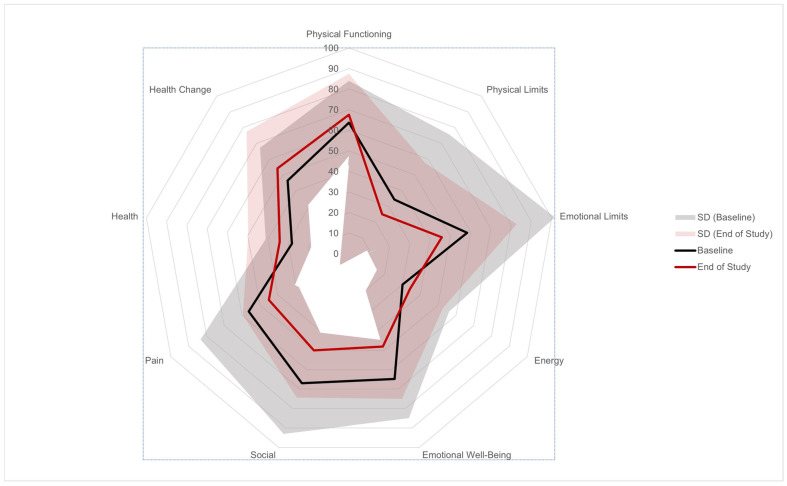
Radar graph of patient quality of life measured using a SF-36 survey. Average score (0–100) of each of the nine domains at baseline (black line) and at week 48 (red line) is shown. Shaded black area represents standard deviation (SD) of each score at baseline while the shaded red area represents SD at week 48. There is no statistical difference between baseline and week 48 at all domains.

Changes of qualities of life over nine domains in the SF-36 survey at week 48 from baseline were shown in Figure 1. A higher score indicates better health. The mean scores for four out of nine domains improved, with the largest improvement observed in the Health Change domain (7.7 points), followed by the Health domain (6.0 points), Energy domain (4.2 points), and Physical Functioning domain (3.8 points). The deteriorations were observed in the Social Functioning, Emotional Well-Being, Emotional Limits, Bodily Pain, and Physical Limits domains (−16.9, −16.8, −12.5, −11.3, and −9.4 points, respectively). The changes in the mean score in all domains were not statistically significant. 

**Figure 2:**
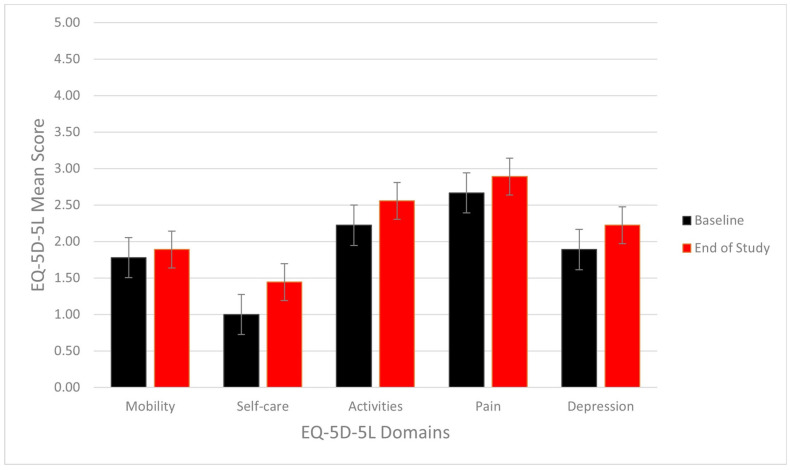
Bar chart comparing EQ-5D-5L mean scores at baseline and at the end of the study. Lower score indicates a better quality of life.

**Figure 3:**
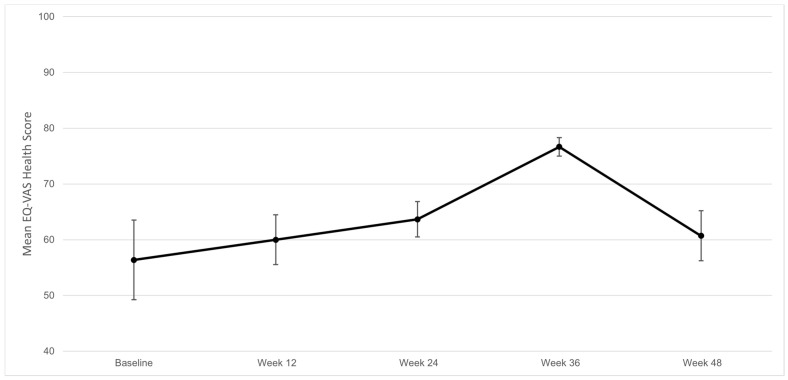
Visual analog scale scores (mean ± SD) of perceived overall health during the 48-week study period. The scale ranges from 0 to 100. “0” represents the worst health one can imagine, whereas “100” represents the best health one can imagine.

The quality of life was also measured using the five dimensions of the EQ-5D-5L survey, shown in Figure 2. There was no statistical difference. An improvement was observed in the mean EQ-VAS score, which increased by 4.34 from baseline to week 48, as shown in Figure 3. This corresponds to an improvement of 7.7%, *p* = 0.23. The mean score reached its peak at week 36, corresponding to a 40.0% increase from baseline.

### 3.5. Serum IgG Level and Infection

The mean serum IgG level at week 48 was comparable to the baseline, 9.6 ± 4.5 vs. 7.6 ± 4.3 g/L, *p* = 0.07. Six patients had an increase in serum IgG level at week 48. 

## 4. Discussion

We reported that 70% of patients in the study who did not previously tolerate 20% SCIG product(s) could tolerate 16.5% SCIG products while maintaining protection from infection and IgG plasma levels. There was no serious AEs related to Ig treatment reported and there was only one injection site infection reported over the 48-week study period. Other AEs were exclusively mild or moderate. Additionally, Cutaquig^®^ SCIG was shown to maintain the quality of life. It is important to note that Cutaquig^®^ was safe and well tolerated during a one-year follow-up despite a larger volume of SCIG product being administered compared to other highly concentrated SCIG products. 

A major challenge of this study is the presence of the Coronavirus Disease 2019 pandemic that overlapped with the study period for 80% of participants. Several patients who completed the week 48 surveys over the phone voiced concerns regarding the impact of the pandemic on their responses for the SF-36 and EQ-5D-5L surveys, indicating that deterioration of their well-being was a result of the pandemic and not their treatment with the Cutaquig SCIG product. The scores pertaining to mental health and emotional well-being decreased during the study period (i.e., emotional well-being, energy, social activities, self-care). Therefore, impacts on the quality of life of patients due to the pandemic were likely reflected in their overall survey scores and may not accurately represent the impact of Cutaquig^®^ on patients’ qualities of life, specifically pertaining to mental health and well-being.

Additionally, the transition to virtual appointments as of March 2020 during the pandemic resulted in missing data. The study was altered to measure the QoL parameters at the baseline and week 48 only, decreasing the quantity of data collected to measure patient satisfaction. The sample size of patients in the study was also small as the number of patients that did not tolerate other SCIG products was low.

## 5. Conclusions

Cutaquig^®^, 16.5% SCIG, offers a tolerable alternative treatment option for patients unable to tolerate 20% SCIG products. Although IG efficacy has been demonstrated to be comparable between brands of SCIG [6], the tolerability could differ due to the viscosity of the product or manufacturing differences. The availability of a 16.5% immunoglobulin treatment expands choices for patients requiring chronic IG treatment. Further research comparing the relative risk of specific common adverse events between Cutaquig^®^ and other 20% SCIGs may offer more insight on treatment options for immunodeficient patients.

## Figures and Tables

**Table 1:** Baseline demographics and immunoglobulin treatment history.

Study ID	Age	Sex	Indication for Ig Rx	Ig Dosage g/Week	Ig Dosage g/kg/Week	Previous Ig Brand	Reason for Intolerability
CQ001	44	M	Primary immunodeficiency (CVID)	10	0.09	Hizentra^®^, Cuvitru^®^, Gammunex^®^, Panzyga^®^, **Gammagard^®^** *	Lower back and joint pain; extreme fatigue
CQ002	44	F	Primary immunodeficiency (CVID)	20	0.17	Hizentra^®^, Gammunex^®^, **Cuvitru^®^**	Severe headache
CQ003	35	F	Primary immunodeficiency (Idiopathic CD4 lymphocytopenia with dysgammaglobulinemia)	10	0.12	Hizentra^®^, **Cuvitru^®^**	Difficulty infusing product-infusion time of 6 h
CQ004	57	F	Secondary immunodeficiency (hypogammaglobulinemia)	8	0.12	Hizentra^®^, Gammunex^®^, **Cuvitru^®^**	Skin rash, extreme fatigue
CQ005	66	F	Secondary immunodeficiency (hypogammaglobulinemia)	10	0.13	Cuvitru^®^	Erythema at injection site, lasting for 1 week post infusion
CQ006	71	F	Primary immunodeficiency (persistent low IgG2 subclass and CD8 T cell count)	8	0.15	Hizentra^®^, **Cuvitru^®^**	Subcutaneous lumps lasting 3 weeks post infusion
CQ007	31	M	Primary immunodeficiency (CVID)	15	0.19	Hizentra^®^, **Cuvitru^®^**	Headache + lower back pain 4 days post infusion, increased infusion time of 3 h
CQ008	73	M	Secondary immunodeficiency (hypogammaglobulinemia)	8	0.08	Hizentra^®^, **Cuvitru^®^**	Restless leg and extreme fatigue
CQ009	52	F	Primary immunodeficiency (combined immunodeficiency)	10	0.12	Cuvitru^®^	Erythema and itchiness at infusion site, shortness of breath
CQ010	48	F	Secondary immunodeficiency (hypogammaglobulinemia)	10	0.10	Hizentra^®^	Headache, nausea, fatigue 2 days post infusion

* administered subcutaneously.

**Table 2:** Cutaquig^®^ infusion parameters at week 48.

Study ID	Ig Dosage g/Week	Infusion Method	Number of Infusion Sites	Volume/Site (mL)	Frequency/Week	Infusion Time (Minutes)	Comments
CQ001	18	Pump	4	27.5	1	120	
CQ002	20	Pump	2	30	2	60	
CQ003	10	Pump	4	12.5	1	90	
CQ004	8	Push	2	20	1	60	
CQ005	10	Pump	1	60	1	Not reported	
CQ006	10	Pump	2	15	1	20	
CQ007	-	-	-	-	-	-	Headache/fatigue 4 days after infusion. Redness around injection site. Discontinued Cutaquig at week 36
CQ008	-	-	-	-	-	-	Restless leg, difficulty sleeping. Discontinued Cutaquig at week 24
CQ009	-	-	-	-	-	-	Itchiness at the injection site post-infusion at week 24 that worsened overtime and discontinued Cutaquig at week 36
CQ010	10	Pump	3	10	2	75	

## Data Availability

The data presented in this study are available in Supplementary Material.

## Supplementary Materials

The following supporting information can be downloaded at: https://www.mdpi.com/article/10.3390/hematolrep14040048/s1, Spreadsheet S1. The study data used for analysis.
